# Interaction between the cellular E3 ubiquitin ligase SIAH-1 and the viral immediate-early protein ICP0 enables efficient replication of Herpes Simplex Virus type 2 *in vivo*

**DOI:** 10.1371/journal.pone.0201880

**Published:** 2018-08-06

**Authors:** Julia S. Czechowicz, Claus-Henning Nagel, Maike Voges, Michael Spohn, Martha M. Eibl, Joachim Hauber

**Affiliations:** 1 Heinrich-Pette-Institute–Leibniz Institute for Experimental Virology, Hamburg, Germany; 2 Biomedizinische Forschungsgesellschaft mbH, Vienna, Austria; 3 German Center for Infection Research (DZIF), partner site Hamburg, Hamburg, Germany; University of St Andrews, UNITED KINGDOM

## Abstract

Herpes Simplex Virus type 2 (HSV-2) is a neurotropic human pathogen. Upon *de novo* infection, the viral infected cell protein 0 (ICP0) is immediately expressed and interacts with various cellular components during the viral replication cycle. ICP0 is a multifunctional regulatory protein that has been shown to be important for both efficient viral replication and virus reactivation from latency. In particular, as previously demonstrated in transfected tissue culture models, ICP0 interacts with the cellular E3 ubiquitin ligase SIAH-1, which targets ICP0 for proteasomal degradation. However, the consequence of this virus-host interaction during the establishment of HSV-2 infection *in vivo* has not yet been elucidated. Here we confirmed that ICP0 of HSV-2 interacts with SIAH-1 via two conserved PxAxVxP amino acid binding motifs. We also demonstrate *in vitro* that a SIAH-1 binding-deficient HSV-2 strain, constructed by homologous recombination technology, exhibits an attenuated growth curve and impaired DNA and protein synthesis. This attenuated phenotype was also confirmed in an *in vivo* ocular infection mouse model. Specifically, viral load of the SIAH-1 binding-deficient HSV-2 mutant was significantly reduced in the trigeminal ganglia and brain stem at day 5 and 7 post infection. Our findings indicate that the interplay between ICP0 and SIAH-1 is important for efficient HSV-2 replication *in vivo*, thereby affecting viral dissemination kinetics in newly infected organisms, and possibly revealing novel targets for antiviral therapy.

## Introduction

Herpes Simplex Virus type 2 (HSV-2) is the causative agent of genital herpes and a ubiquitous human virus present in about 10–20% of the human population in Europe and the US [1]. HSV-2 initially infects and replicates in epithelial cells and invades the nervous system by entering the innervating sensory neurons [2]. The virus then undergoes retrograde transport along the axon to the neuronal cell bodies, where a lifelong latency is established [2,3]. Due to a variety of stimuli, HSV can occasionally reactivate from its latent state. In this case, the virus replicates in the neuron and is transported anterograde to or near the side of primary infection to re-infect epithelial cells [2]. While HSV-2 is mostly benign in hosts with an intact immune system, it can cause severe disease in immunocompromised patients, for instance when the central nervous system is infected, or when HSV is transmitted to a newborn during birth [4–6]. Moreover, it has been reported that genital HSV infection significantly increases the risk of HIV-1 infection [7].

HSV gene expression is regulated by host and viral transcription factors and takes place in a cascade-like manner [5,8]. One of the first viral genes transcribed is the immediate early gene *RL2*, which encodes infected cell protein 0 (ICP0). ICP0 is a multifunctional *trans*-activator of viral gene expression during lytic infection [9–16] and is said to play a role in reactivating the virus from latency [15,17–19]. ICP0 acts as an E3 ubiquitin ligase through its N-terminal RING domain, an activity essential for many functions ascribed to ICP0 [9,20]. Thus, ICP0-mediated polyubiquitination leads to proteasomal degradation of cellular targets [9,12]. Examples are the ICP0-mediated degradation of SUMOylated isoforms of PML and Sp100 in the host cell nucleus that occurs shortly after HSV infection [12,21–24].

The importance of ICP0 for the virus can be further demonstrated with ICP0-deficient HSV mutants. These mutants are severely defective in establishing infection and high MOIs are necessary for these mutant viruses to overcome intrinsic antiviral cellular defenses [9,12,25–29]. Therefore, studying the interaction of ICP0 with cellular targets is crucial to understand the molecular pathology of HSV-2 and to elucidate possible novel targets for antiviral therapy.

Previously, we described a novel cellular interaction partner of ICP0, the human E3 ubiquitin ligase *seven in absentia homolog 1* (SIAH-1) [30]. Initially identified as *seven in absentia* (SINA) in Drosophila, the family of SIAH proteins contains two human homologs, SIAH-1 and SIAH-2 [31]. Although they differ at their N-terminus, both human SIAH homologs share a RING domain that confers E3 ubiquitin ligase activity, two zinc-finger domains, as well as a C-terminal substrate-binding domain [32,33].

Here, we investigated the effect of the ICP0:SIAH-1 interaction on HSV-2 infection and replication *in vitro* and *in vivo* employing various mutants generated by homologous recombination from the parental HSV-2 strain MS. Our results revealed that SIAH-1 binding-deficient HSV-2 strains are growth attenuated and synthesize significantly less that viral protein and DNA, indicating that the ICP0:SIAH-1 interaction is required for efficient HSV-2 replication *in vivo*.

## Materials and methods

### Cell lines

African Green monkey kidney epithelial cells (Vero; ATCC#: CCL-81), baby hamster kidney epithelial cells (BHK-21, ATCC CCL-10), human embryonic kidney cells (HEK293T; ATCC#: CRL-11268), and human osteosarcoma cells (U2OS; ATCC#: HTB-96) were cultured in DMEM supplemented with 10% fetal calf serum (FCS), 2 mM L-glutamine, 1 mM sodium pyruvate, and 3.75 mg/ml sodium bicarbonate. Human hepatic cells (HepaRG^TM^; Life Technologies, Carlsbad, CA) were cultured in full DMEM supplemented with 5 μg/ml bovine insulin and 50 μM hydrocortisone.

### Mouse strains

C57Bl/6J mice were originally obtained from the Jackson Laboratory and bred and maintained under specific pathogen-free (SPF) conditions at the animal facility of the Heinrich-Pette-Institute, Hamburg. Animals were daily monitored for signs of obvious suffering, such as weight loss, back arches and prostrated behavior.

Animal experiments were performed according to the guidelines of the German Animal Protection Law. The experimental protocols were reviewed and approved by the relevant German authority, the Freie und Hansestadt Hamburg, Behörde für Gesundheit und Verbraucherschutz (Nr.: 144/15).

### Viruses

Unmodified plaque purified [34] HSV-2 strain MS (original non-purified virus received from ATCC#: VR-540) served as the initial virus for homologous recombination. Construction of HSV-2 ­ICP0^GFPΔ19­162^ (HSV-2 0ΔRING) is described elsewhere [28].

### Reagents and antibodies

Protein G sepharose and glutathione sepharose were obtained from GE Healthcare (Freiburg, Germany). GFPtrapA beads comprise a single domain GFP antibody immobilized on agarose beads (Chromotek, Martinsried, Germany). Proteasome inhibitor MG132 was purchased from Sigma (Munich, Germany). The following antibodies were used: anti-tubulin, mouse IgG (Sigma); anti-GFP (Novus Biologicals, CO); anti-PML H-238, anti-SIAH-1 N-15 (Santa Cruz), anti-HSV1/2 VP5 H1.4 (Acris, Herford, Germany); anti-ICP0 R147 and anti-ICP27 R152 (Biomedizinische Forschungsgesellschaft mbH Vienna, Austria), and anti-GST-DX700 (IRDye® labeled antibodies; Rockland Immunochemicals, Gilbertsville, CA). For infrared fluorescence detection (Li-COR Biosciences, Bad Homburg, Germany) secondary antibodies coupled to IRDye® fluorophores (Rockland) were used. Glutathione sepharose for GST-pulldown experiments was obtained from GE Healthcare (Freiburg, Germany).

### Expression constructs

A eukaryotic expression plasmid encoding a codon-optimized and intron-less HSV-2 ICP0 sequence with a 3’ in-frame fusion to the coding sequence of green fluorescent protein (eGFP), as well as an expression plasmid encoding ICP0 with a deletion of the RING domain (ΔRING, Δaa126-166) were described previously [30]. Using the QuikChange site-directed mutagenesis kit (Agilent), point mutations in the two SIAH binding motifs were introduced using following oligonucleotide primers: 5’-GCACGTCCACGCGCTGCTAATGCAAATCGCGTGC GCTCTCCACC-3’ and 5’-GGTGGAGAGCGCACGCGATTTGCATTAGCAGCGCGTGGACGTGC-3’ for the SIAH binding motif at aa position 414–416 (NxN1); 5’-GGCCCAGCAGTGGCAGCTAAT GTGAATCGTGTGGCATCTCTGCCAC-3’ and 5’-GTGGCAGAGATGCCACACGATTCACATTAGCT GCCACTGCTGGGCC-3’ for the motif at aa position 316–318 (NxN2). Eukaryotic expression plasmids encoding HA-SIAH-1, HA-SIAH-1^C44S^, as well as a plasmid for bacterial expression of GST-SIAH-1 were described elsewhere [30,35].

### Plasmid precursors of HSV-2 recombinant viruses

To construct the SIAH-binding deficient HSV-2 ICP0 mutants, transfer plasmids were generated. First, a homology plasmid was constructed by subcloning two DNA fragments derived from wild-type HSV-2 MS genomic DNA into a pUC19 plasmid vector (New England Biolabs), encompassing sequences homologous to 517 bp upstream of the HSV-2 ICP0 start codon and 545 bp downstream of the ICP0 stop codon. During cloning, XbaI and BamHI restriction sites were introduced in-between the two homology arms. Next, pICP0-GFP plasmids (see above) and the homology plasmid were treated with the enzymes XbaI and BamHI and fragments were separated via gel electrophoresis. The linearized homology plasmid as well as the XbaI-BamHI DNA fragment (ICP0-GFP sequence) from the pICP0-GFP plasmids, were extracted and the ICP0-GFP sequence was ligated into the homology plasmid using the DNA Rapid Dephosphorylation and Ligation Kit (Roche). The resulting transfer plasmids, where the wild-type or mutated ICP0-GFP sequence is flanked by the homology arms, are shown in S1 Table.

### HSV-2 mutagenesis by homologous recombination

U2OS cells were transfected with 2.5 μg of the linearized transfer plasmid. After 24 hours, transfected cells were infected with plaque-purified HSV-2 strain MS at an MOI of 5 pfu/cell. Recombinant viruses were identified by GFP fluorescence and isolated via plaque picking and up to four cycles of subsequent plaque purification by limiting dilution [34]. Prior to further characterization, two independent recombinant viruses were routinely isolated from parallel experiments.

### DNA sequencing

The sequences encoding ICP0-derived SIAH binding motifs in the various constructs were amplified by using following oligonucleotides: forward primer, 5’-GATGAAGATGATGACCTG-3’; reverse primer, 5’-AAGATGCAGAACTAGACG-3’. The resulting 850 bp amplification products were ligated into a TOPO vector using the TOPO PCR cloning kit (Life Technologies) and transformed into the bacterial strain DH5α. Selected clones were sequenced with standard M13 forward and reverse primers to exclude PCR-derived point mutations as false negatives. Positive mutants were subsequently transfected into U2OS cells and plaque-purified three to four times.

The integrity of HSV-2 genomes was verified by next generation sequencing. For this, purified viral DNA isolated from infected BHK cells was sheared by sonification. Library preparation was conducted according to the *Protocol for use with NEB Next Ultra DNA Library Prep Kit for Illumina (E7370)* (NEB) and libraries were sequenced using a MiSeq (Illumina) device. SPAdes v3.10.1 was used for in silico assembly of the respective sequencing reads, which were subsequently mapped to the reference HSV-2 strain HG52 (NC_001798.2) using the blast v2.7.1.

### DNA transfection

U2OS cells were transfected using TransIT (Mirus) according to the manufacturer’s protocol. HEK293T cells were transfected using Polyethylenimine (PEI; Polysciences, Eppelheim, Germany) at a mass ratio of PEI and DNA of 6:1.

### Virus production

BHK-21 cells (ATCC#: CCL-10) were used for preparation of virus stocks from infected cell culture supernatants as described previously [36,37]. Virus stocks of passage 2 or 3 were used for all subsequent experiments.

### Quantification of viral DNA

Viral DNA was isolated from infected cell pellets using the Qiagen Blood Mini Kit (Hilden, Germany) or from infected tissue using the peqGOLD TriFast reagent (Thermo Fisher Scientific, Waltham, MA) and DNA was quantified by Taqman real-time PCR (Applied Biosystems 7500 Real-Time PCR System; Life Technologies) using oligonucleotide-primers specific for the HSV-2 *U*_*L*_*27* gene sequence encoding glycoprotein B [38] and for the GFP-sequence as follows: *U*_*L*_*27*-forward primer, 5’-TGCAGTTTACGTATAACCACATACAGC-3´; *U*_*L*_*27*-reverse primer, 5´-AGCTTGCGGGCCTCGTT-3´; *U*_*L*_*27*-probe, 5’-FAM-CGCCCCAGCATGTCATTCACGT-TAMRA-3´; GFP-forward primer, 5´-ACAAGCTGGAGTACAACTA-3´; GFP-reverse primer, 5’-TGTTCTGCTGGTAGTGGTC-3’; and GFP-probe, 5’-FAM-CAGCCACAACGTCTATATCATG-TAMRA-3´. In human cell lines, viral DNA copies were normalized to sequences of the human beta-globin (HBG) gene [39]; HBG-forward primer, 5’-CTTAATGCCTTAACATTGTGTATAA-3’; HBG-reverse primer, 5’-GAATATGCAAATAAGCACA CATATAT-3’; HBG-probe, 5’-FAM-ACTTTACACAGTCTGCCTAGT ACATTAC-TAMRA-3’. In murine cells (tissue), viral DNA copies were normalized to beta-actin (BAK) gene sequences. BAK-forward primer 5´-TCCTGAGACTCCCAGCACAC-3´; BAK-reverse primer, 5’-ACACTCAG GGCAGGTGAAACT-3´; BAK-probe, 5’-FAM-TGCACTCCTTGCAT GTCTCAGA-TAMRA-3’.

### Southern blot analysis

Viral DNA was digested using the restriction endonucleases HindIII and NcoI. DNA fragments were separated via standard gel electrophoresis and transferred to nitrocellulose membranes by capillary blotting. The utilized probes were directed against a 726 bp sequence in GFP and against a 860 bp sequence within HSV-2 wild-type ICP0 that did not recognize the codon-adjusted ICP0-GFP sequence in the recombinant viruses. Probes were synthesized via PCR using following GFP-specific oligonucleotide-primers: forward, 5’-ATTTACTCGAGGCTAGCATGGCCACAACCATGGTGAG-3’ containing an XhoI and NheI restriction site; reverse, 5’-TAAATCTCGAGTTACTTGTACAGCTCGTCCATG-3’ containing an XhoI restriction site. The wild-type ICP0 primers used were as follows: forward, 5’-ATTTACTGCAGCACGGTGAGAGGGCGA-3’; reverse, 5’-ATTTACTGCAGACTATCAGGTACGC CACC-3’ both with PstI restriction sites. Probes were radioactively labelled with ^32^P-dCTP (Hartmann Analytic, Braunschweig, Germany). Hybridization was conducted using the ExpressHyp hybridization solution (TAKARA/CLONTECH). After hybridization, the membrane was subjected to autoradiography.

### Protein-protein interaction analysis

Preparation of cell lysates from transfected cells and immunoprecipitation using GFPtrapA beads or Protein G Sepharose-coupled antibodies were performed as described previously [30]. For infected cell lysates, U2OS cells were infected with wild-type HSV-2 strain MS, HSV-2-ICP0-GFP or the respective SIAH binding-deficient ICP0-GFP mutants at an MOI of 0.01 pfu/cell. At 48 hours post infection, cells were harvested, washed with PBS, centrifuged at 20,000 x g for 20 sec and resuspended in 650 μl RIPA lysis buffer +1x protease inhibitors. Lysates were incubated on ice for 30 min and afterwards centrifuged at 20,000 x g for 15 min at 4°C. 10% of the supernatant was resuspended in 10 μl 6x protein sample buffer (PP) (350 mM Tris/HCL pH 6.8, 10% [w/v] SDS, 36% [v/v] glycerol, 6% [v/v] beta-mercaptoethanol, 0.01% [w/v] bromophenol blue in distilled water) as control and the cell pellet was resuspended in 650 μl 1x PP.

For GST-pulldown assays, *E*.*coli* BL21(DE3)pLysS (Promega), carrying pGEX-SIAH-1 or the empty GST plasmid pGEX-5T were grown overnight in LB medium at 37°C. The next day, 250 ml LB medium with 100 μg/ml ampicillin were inoculated with 100 μl of overnight culture and shaken at 37°C until the OD600 reached 0.5. For induction, IPTG was added at 1 μg/ml (GST-SIAH-1) or 5 ng/ml (GST) and the cultures were shaken further at 30°C for 4 h, 0.1 μM ZnCl_2_ were included in the GST-SIAH-1 culture during induction. The cell pellets were lysed for 10 min on ice with 3 mg/ml lysozyme in 50 mM Tris-Cl, pH 8.0, 150 mM NaCl, 5 mM EDTA, 1% TX-100, sonicated and centrifuged at 20,000 × g for 30 min at 4°C. The lysates were shaken for 30 min at room temperature with 1 ml glutathion sepharose beads equilibrated in NETN buffer (20 mM Tris-HCl pH 8.0, 100 mM NaCl, 1 mM EDTA, 0.4% NP-40). For preclearing, the GST-loaded beads were washed three times with NETN and then added to the cell lysates containing the potential SIAH interacting proteins and shaken on an overhead mixer for 1 h at 4°C. Afterwards, the beads were removed by pelleting and the precleared lysates were incubated with GST-SIAH-1 coupled beads overnight at 4°C. The beads were washed three times with 50 mM HEPES pH 7.5, 150 mM NaCl, 5 mM EDTA, 0.5% (v/v) NP-40 and the pellets were resuspended in 2×SDS-PAGE sample buffer (see below).

### Western blot analysis

For preparation of cellular extracts, cells were washed with PBS and dissolved in 2xSDS-PAGE sample buffer (116 mM Tris-HCl pH 6.8, 3.3% [w/v] SDS, 12% [v/v] glycerol, 2% [v/v] beta-mercaptoethanol, bromophenol blue) and 1x complete cOmplete™ protease inhibitor cocktail (Roche). After separation by SDS-PAGE, proteins were transferred to nitrocellulose membranes (Schleicher & Schuell) and subjected to immune blot analysis.

### Virological assays

Standard plaque assays were conducted in a 6-well format to determine the titers (plaque formation efficiency) of wild-type and mutant HSV-2. Duplicate dilution series were set up in RPMI (RPMI 1640 containing 10% [v/v] FCS) and were used to inoculate confluent U2OS, HepaRG or Vero cells. After 1 h of incubation on a rocking platform, the inoculum was removed and cells were covered with 2 ml complete DMEM containing 25 μg/ml HSV neutralizing human polyclonal IgG (Sigma-Aldrich, St. Louis, MO) and incubated at 36°C and 5% CO_2_. Procedures were according to the protocol in [40]. After 48 hours, culture medium was removed and cells were fixed with 0.5 ml/well ice cold methanol and stained with 0.1% crystal violet.

### Ocular infection of mice

Six to eight week old female C57Bl/6J mice were anesthetized by intraperitoneal (i.p.) injection of ketamine (100 mg/kg of body weight) and xylazine (5 mg/kg of body weight). Each cornea was scarified in a crosshatch pattern with a 26-gauge needle and subsequently inoculated with 5x10^5^ plaque forming units (PFU) of HSV-2-ICP0-GFP or HSV-2-ICP0^NxN1/2^-GFP strain MS, or with DMEM as control. The virus was suspended in 4 μl of complete cell culture medium and administered as an eye drop. Viral titers were determined at the indicated time points post inoculation on Vero cell monolayers.

### Determination of viral titers in the tear film and tissue of C57Bl/6J mice

For analysis of ocular virus shed, tear film swabs were taken from both eyes on day 1, 2, 3, 5 and 7 post infection using a cotton-tipped applicator. Each tip was placed in 0.4 ml of complete cell culture medium, vortexed and surplus liquid was removed by squeezing. The amount of virus was determined by a microtiter plate plaque assay (96-well format) on Vero cell monolayers [41].

Mice were euthanized and the trigeminal ganglia (TG) and brain stem were isolated and placed in 0.6 ml of complete cell culture medium on day 3, 5 and 7 post infection. TGs from the left and the right eye were pooled. The isolated tissues were homogenized with a sonifier (Branson B-12) and freeze/thawed afterwards. The cell debris was removed by centrifugation at 3,000 x g for 5 min and the supernatant was taken for virus titer determination using standard plaque assay on Vero cell monolayers.

### Statistical tests

GraphPad Prism 5.03 was used to perform statistical analysis for the qRT-PCR data and viral growth analysis *in vitro* and *in vivo*. For *in vivo* analysis, mean ± standard error of the mean (SEM) are reported for each group. T-test was used for two-group comparison with Mann-Whitney post-test when Gaussian distribution was not assumed. 2-way analysis of variance (ANOVA) with Bonferroni post-test was used to compare multiple groups and time points and replicate means. Significance was determined with p-values <0.001 defining *** significance and p-values >0.05 defining no significance.

## Results

### ICP0 interacts with SIAH-1 via two minimal interaction motifs

**Fig 1:**
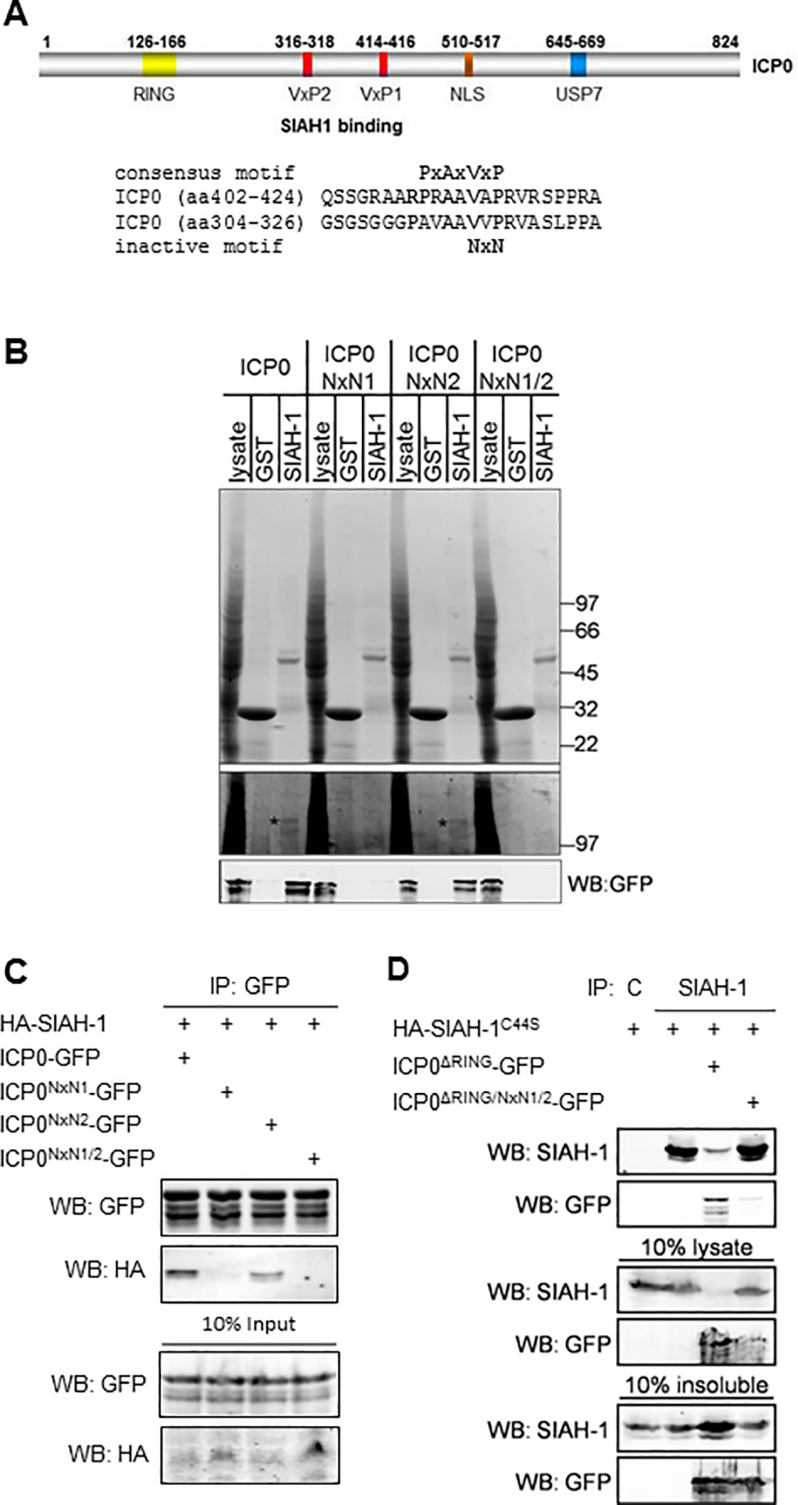
ICP0 interacts with SIAH-1 via two minimal interaction motifs. (**A**) Schematic representation of HSV-2 ICP0 indicating the position of the RING domain (yellow), the USP7 interaction domain (blue), the nuclear localization signal (brown) and the two SIAH interaction motifs VxP1 and VxP2 (red). The primary amino acid sequence surrounding the predicted SIAH binding motifs is depicted together with the consensus motif and the position of the inactivating NxN mutation. (**B**) HEK293T cells were transfected with plasmids encoding GFP-tagged ICP0 and its mutants and the cell lysates were incubated with GST or GST-SIAH-1-loaded glutathione sepharose beads. The upper SDS-PAGE gel shows a Coomassie staining of the respective input control (lysate) and the eluates from the GST or GST-SIAH-1 beads. Below, a contrast enhanced section of the gel with putative ICP0 bands indicated by asterisks. ICP0 was detected by Western blotting using an antibody against the C-terminal GFP tag. Size markers in kDa. (**C**) HEK293T cells were transfected with plasmids encoding GFP-tagged ICP0 and its mutants and HA-tagged SIAH-1. The ICP0-GFP proteins were immunoprecipitated from the lysates, ICP0 and SIAH-1 were detected by SDS-PAGE and Western blotting using antibodies against the GFP and HA-tags. (**D**) HEK293T cells were transfected with plasmids encoding the inactive mutant HA-SIAH-1^C44S^ and GFP-ICP0^ΔRING^ or GFP-ICP0^ΔRING/NxN1/2^ as indicated. Immunoprecipitation from the cell lysates was performed using control mouse IgG or anti-SIAH-1. ICP0 and SIAH-1 were detected by SDS-PAGE and Western blotting using antibodies against SIAH-1 or the GFP-tag. The lower panel shows the analysis of the RIPA buffer-insoluble pellet after cell lysis.

Previously, we identified a SIAH-1 binding consensus motif PxAxVxP in HSV-2 ICP0 [30]. Its most important core residues are located at ICP0 amino acid positions 414–416 (VxP1; Fig 1A). Co-immunoprecipitation studies with several ICP0 truncation and internal deletion mutants demonstrated that deletion of residues 410–420 greatly reduced the ICP0:SIAH-1 functional interaction. We also identified a second putative SIAH-1 binding motif around residues 316–318 (VxP2, Fig 1A), which does not perfectly overlap with the consensus PxAxVxP sequence. In many proteins targeted by SIAH ubiquitin ligases, a VxP motif is essential for efficient binding and mutation of the core residues to NxN inactivates SIAH-binding [42,43]. Therefore, to analyze *in vitro* whether a NxN mutation is sufficient to abolish the ICP0:SIAH-1 interaction, we first introduced point mutations into a previously described HSV-2-ICP0-GFP expression plasmid [30].

ICP0-GFP or its NxN mutants were transiently expressed in HEK293T cells and the cell lysates were incubated with GST-SIAH-1-loaded glutathione sepharose beads, or beads loaded with GST alone as a control. Pulldown assays and subsequent Western blotting demonstrated that ICP0 and ICP0^NxN2^ were bound to SIAH-1-loaded beads (Fig 1B, lower panel), whereas ICP0^NxN1^ and the double mutant ICP0^NxN1/2^ were no longer bound. Subsequently, the ICP0:SIAH-1 interaction was confirmed by immunoprecipitation of GFP from lysates of cells co-transfected with hemagglutinin tagged SIAH-1 (HA-SIAH-1) and ICP0-GFP (Fig 1C). Consistent with the previous experiment, ICP0, and to a lesser extent ICP0^NxN2^, bound to SIAH-1, while ICP0^NxN1^ binding was barely detectable and ICP0^NxN1/2^ showed no binding. In reverse, SIAH-1 immunoprecipitation confirmed the loss of interaction of the ICP0^NxN1/2^ mutant (Fig 1D). In this experiment we employed RING-deficient ICP0 and SIAH-1 mutants to increase protein stability. Less SIAH-1 was present in the cell lysates co-transfected with ICP0^ΔRING^ than in the absence of ICP0 or when ICP0^ΔRING/NxN1/2^ was present. This might be due to the formation of buffer-insoluble SIAH-1:ICP0 aggregates (Fig 1D, lower panels).

In summary, mutation of the two VxP core motifs resulted in reduced binding of HSV-2 ICP0 to SIAH-1. The affinity of the VxP1 motif appears to be stronger than that of the VxP2 motif.

### Construction and validation of SIAH-binding deficient ICP0 mutant virus

**Fig 2:**
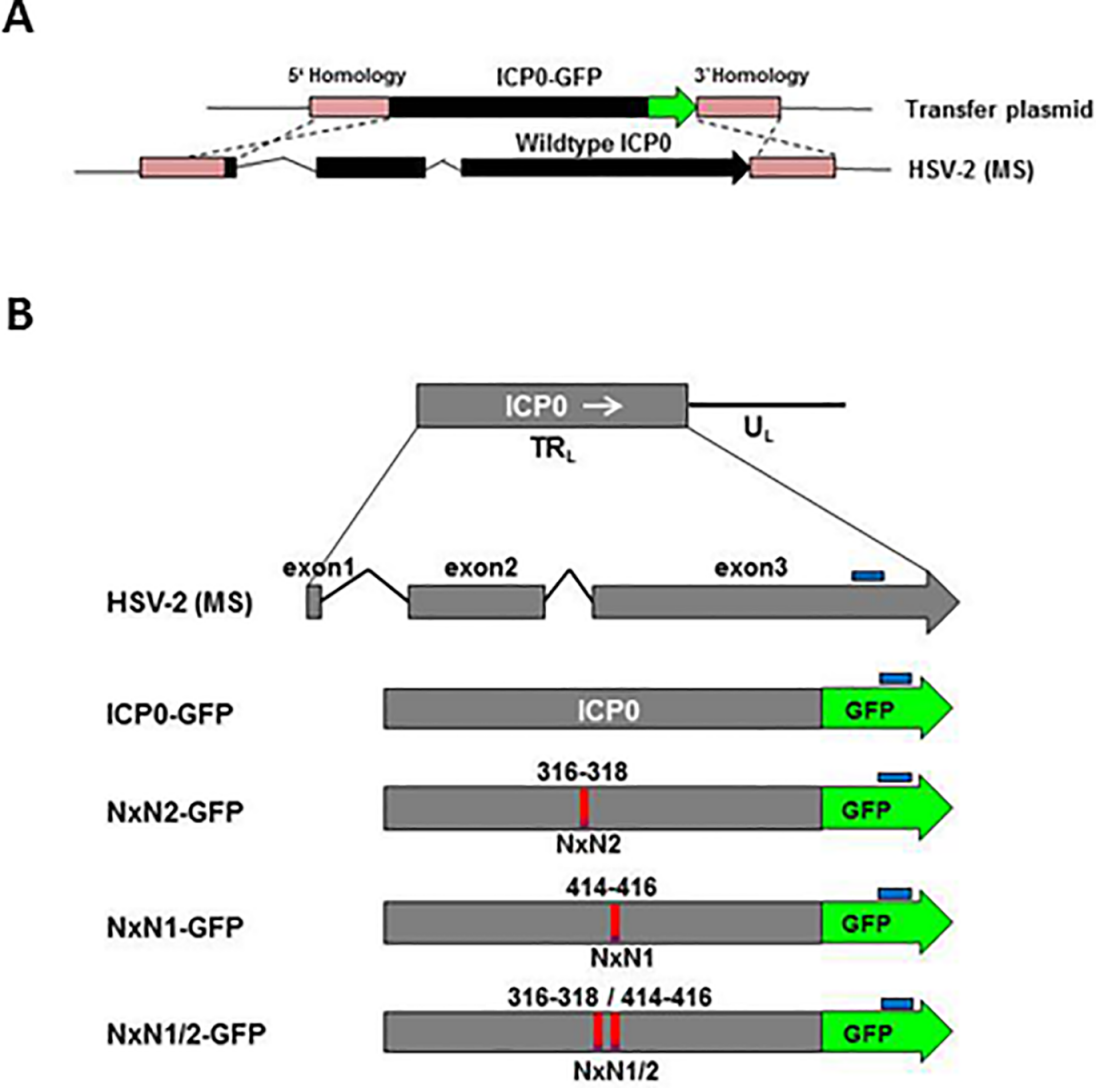
Construction of HSV-2 mutant viruses with mutated SIAH binding motifs in ICP0. (**A**) Depiction of homologous recombination. The native ICP0 sequence in HSV-2 strain MS was exchanged by a sequence encoding ICP0-eGFP fusion protein. (**B**) Maps of the various ICP0 constructs. The wild-type gene is located in the HSV-2 TR_L_ region. The various ICP0-GFP DNA sequences were accordingly adjusted to reduce their GC content. The NxN1 and NxN2 mutations in the SIAH-1 binding domains are marked in red. Sequences recognized by Southern blot probes are marked in blue.

To analyze the relevance of the ICP0:SIAH-1 interaction in the context of viral infection we introduced the VxP1 and/or VxP2 mutations (Fig 1A) into both ICP0 coding regions of a full-length HSV-2 genome. HSV-2 mutants with ICP0 genes additionally fused 3’ in-frame to the GFP coding region were generated by homologous recombination (Fig 2A; for list of transfer plasmids see S1 Table). The viruses expressing either wild-type ICP0-GFP or ICP0-GFP carrying mutated VxP core residues in the two SIAH binding domains, either individually mutated (NxN1 or NxN2) or mutated in combination (NxN1/2; Fig 2B) were analyzed by restriction endonuclease digestion and subsequent Southern blot analysis (S1 Fig). Furthermore, mutations, as well as overall genome integrity were confirmed by Illumina MiSeq™ sequencing of the full-length viral constructs.

Subsequent *in vitro* control experiments revealed that no significant growth differences exist between wild-type HSV-2 strain MS and HSV‐2‐ICP0‐GFP (S2 Fig). Furthermore, wild-type and mutant constructs (i.e. HSV-2-ICP0-GFP and HSV-2-ICP0^NxN1/2^-GFP, respectively) displayed comparable phenotypes with respect to PML degradation (S3 Fig) and timely translocation of ICP0 to the cytoplasm (S4 Fig).

### Mutation of ICP0 in HSV-2 abolishes SIAH-1 binding

**Fig 3:**
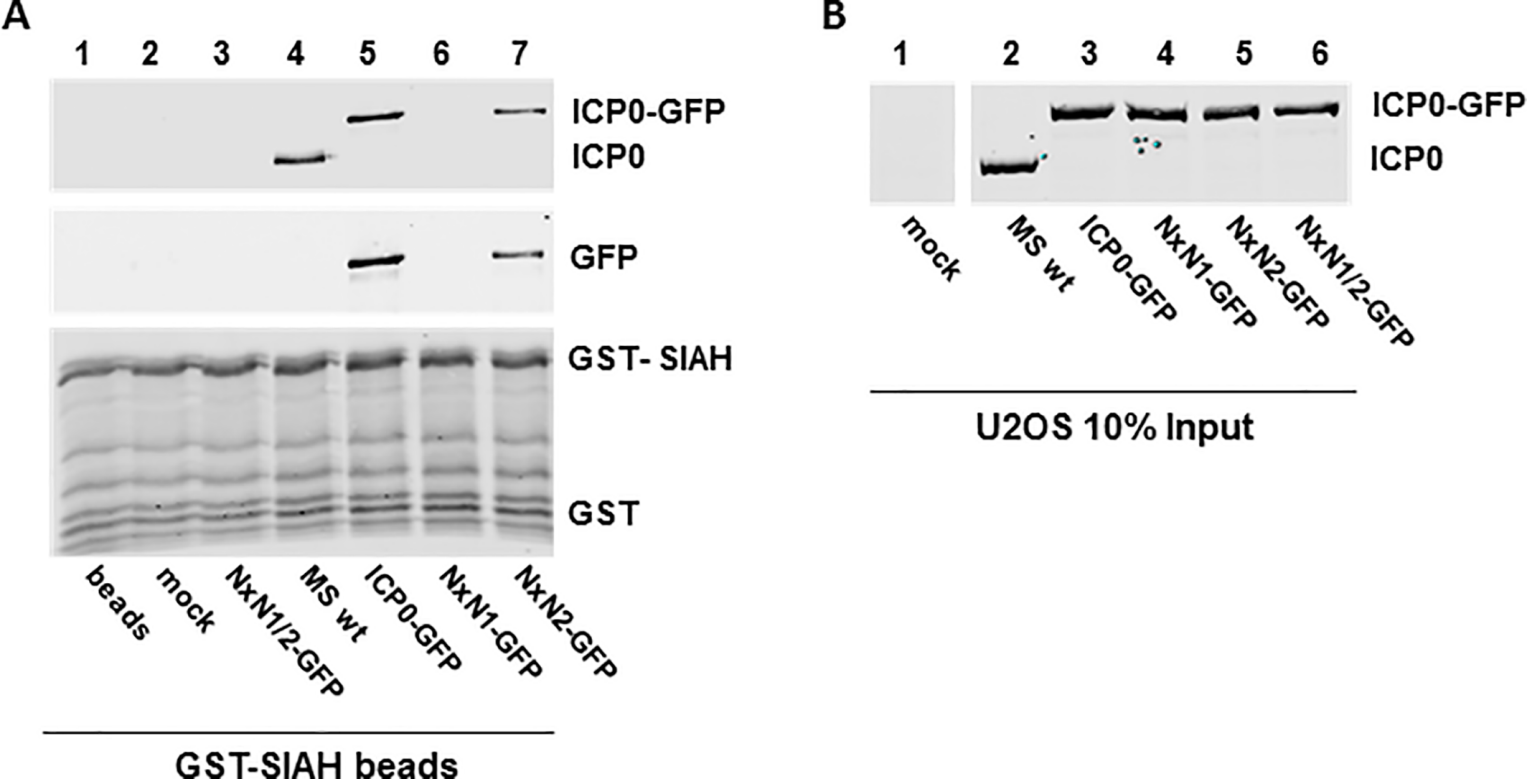
Virally expressed ICP0^NxN1/2^ does not bind to SIAH-1. (**A**) U2OS cells were infected for 48 h with the indicated mutants and HSV-2 strain MS (MS wt) at an MOI 0.01 pfu/cell. Cell lysates were incubated with GST-SIAH-1-loaded glutathione sepharose beads. Eluates were analyzed by SDS-PAGE and Western blotting using antibodies directed against HSV-2 ICP0 and GFP. (**B**) Input controls (10%) of the GST-pulldown were analyzed as before using ICP0-specific antibody.

To test SIAH-1 binding efficiency by the various viruses, U2OS cells were infected for 48 hours with an MOI of 0.01 pfu/cell before performing GST pull-down assays (Fig 3). Cell lysates were harvested and incubated with GST-SIAH-1-loaded glutathione sepharose beads. ICP0 expressed by wild-type HSV-2 strain MS (MS wt) or the HSV-2-ICP0-GFP (ICP0-GFP) clearly bound to GST-SIAH-1 loaded beads, whereas the mutated HSV-2-ICP0^NxN1/2^-GFP (NxN1/2-GFP) showed no detectable binding (Fig 3). Interestingly, ICP0 still interacted with SIAH-1 when the VxP2 binding motif was disrupted, although the binding affinity seemed weaker than with wild-type ICP0. In agreement with our transfection experiments, inactivation of the VxP1 motif was sufficient to almost completely abolish SIAH-1-binding in these *in vitro* experiments, suggesting that particularly the VxP1 motif is critical for the interaction between HSV-2 ICP0 and cellular SIAH-1.

### The NxN1/2 mutation in ICP0 attenuates viral growth

Compared to wild-type HSV, significantly fewer plaques are produced by ICP0null mutants on many cell lines, including Vero cells [16,26,27]. In sharp contrast, deletion of ICP0 does not affect plaque formation in U2OS cells [38,44,45]. However, the previously reported RING-finger-deficient HSV-2-GFP-ICP0^Δ19–162^ (HSV-2 0ΔRING) mutant [28] produced severely reduced vital titers, by almost two logs, on Vero cells compared to U2OS cells (S2 Table and S5 Fig). However, plaque formation of the SIAH-1 binding-deficient HSV-2 ICP0 mutant was clearly not affected on these cell lines (S2 Table and S6 Fig). Notwithstanding, during preparation of virus stocks we noted delayed growth of our HSV-2-ICP0^NxN1/2^-GFP mutant. To confirm this observation, we compared the growth kinetics of HSV-2-ICP0^NxN1/2^-GFP and HSV-2-ICP0-GFP in U2OS and Vero cells using an MOI of 1 pfu/cell. Again, HSV-2-GFP-ICP0^Δ19–162^ was included as a control in some experiments. Of note, analysis of HSV-2-ICP0^NxN1/2^-GFP and HSV-2-ICP0-GFP particle preparations by major capsid protein VP5-specific immunoblotting and *UL27*-specific droplet digital PCR demonstrated comparable virion/pfu ratios (not shown).

**Fig 4:**
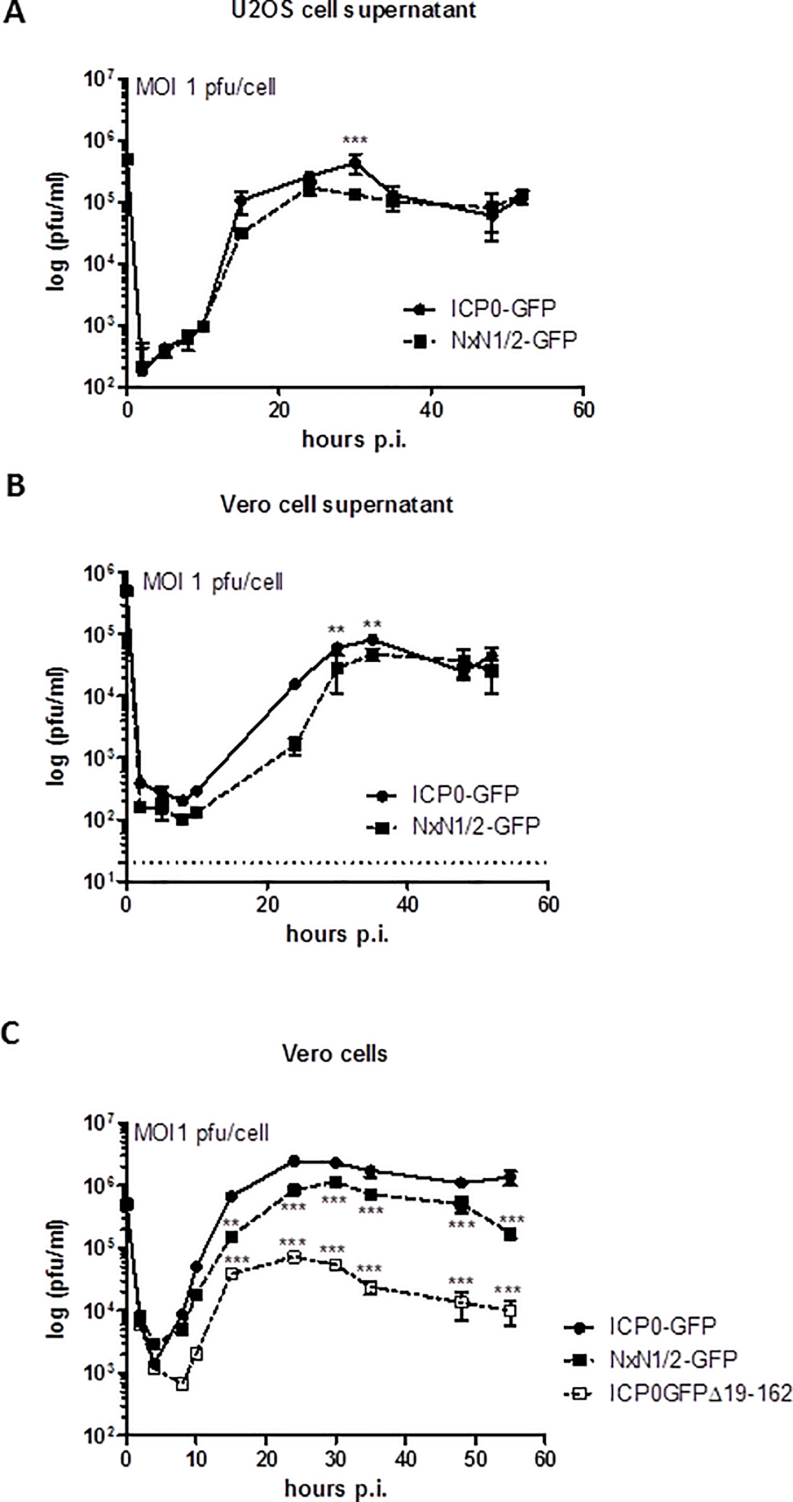
The NxN1/2 mutation in ICP0 of HSV-2 results in delayed viral growth. (**A**) U2OS cells were infected in duplicates with an MOI of 1 pfu/cell of HSV-2-ICP0-GFP (ICP0-GFP) and HSV-2-ICP0^NxN1/2^-GFP (NxN1/2-GFP). At the indicated time points supernatants were harvested and titrated on Vero cell monolayers. Mean ± SD of viral titers (n = 2 per data point) were plotted as a function of time. (**B**) Vero cells were infected in duplicates and evaluated as above. (**C**) Vero cells were infected in duplicates with HSV-2-ICP0-GFP, HSV-2-ICP0^NxN1/2^-GFP and HSV-2-GFP-ICP0^Δ19–162^ (i.e. ICP0ΔRING mutant) at an MOI of 1 pfu/cell. At the indicated time points supernatants were harvested, titrated and evaluated as above. Titer differences were analyzed using two-way ANOVA, with ** p<0.01 and *** p<0.001. The detection limits are shown by a dashed line.

After infecting U2OS or Vero cells, supernatants were harvested at regular intervals for subsequent titration using Vero cell monolayers (Fig 4A and 4B). In another set-up, infected Vero cell cultures were lysed and titrated (Fig 4C). Independently of the procedure and cell line used, the titers of the ICP0^NxN1/2^-GFP mutant were significantly lower at 30 to 35 hours post infection than the titers of HSV-2-ICP0-GFP. By about 40 to 48 hours post infection, both viruses reached approximately comparable titers when analyzing virus-containing supernatants (Fig 4A and 4B). However, when viral particles isolated from Vero cell pellets were analyzed, the observed growth differences between the NxN1/2 mutant and HSV-2-ICP0-GFP were more pronounced (Fig 4C). At 55 hours post infection, when this experiment was terminated, the titer of the SIAH-1 binding-deficient mutant (NxN1/2) was clearly lower than the ICP0-GFP virus titer. Indeed, titer differences between the two viruses were already significantly different at about 15 hours and increased at 25 hours post infection.

As expected, the RING-finger-deficient mutant (i.e. HSV-2-GFP-ICP0^Δ19–162^) produced significantly diminished viral titers, also starting at 15 hours post infection (Fig 4C). By 35 hours post infection, the titers of this ΔRING mutant were more than one log below the titer of the NxN1/2-GFP mutant and almost two logs below the titer of wild-type virus (Fig 4C). These results suggest that preventing interaction between ICP0 and SIAH-1 attenuates growth of HSV-2, although clearly this growth disadvantage is not as striking as the reduced growth behavior of the RING-finger-deficient (ICP0ΔRING) mutant (Fig 4C).

### The NxN1/2 mutation impairs viral protein and DNA synthesis *in vitro*

**Fig 5:**
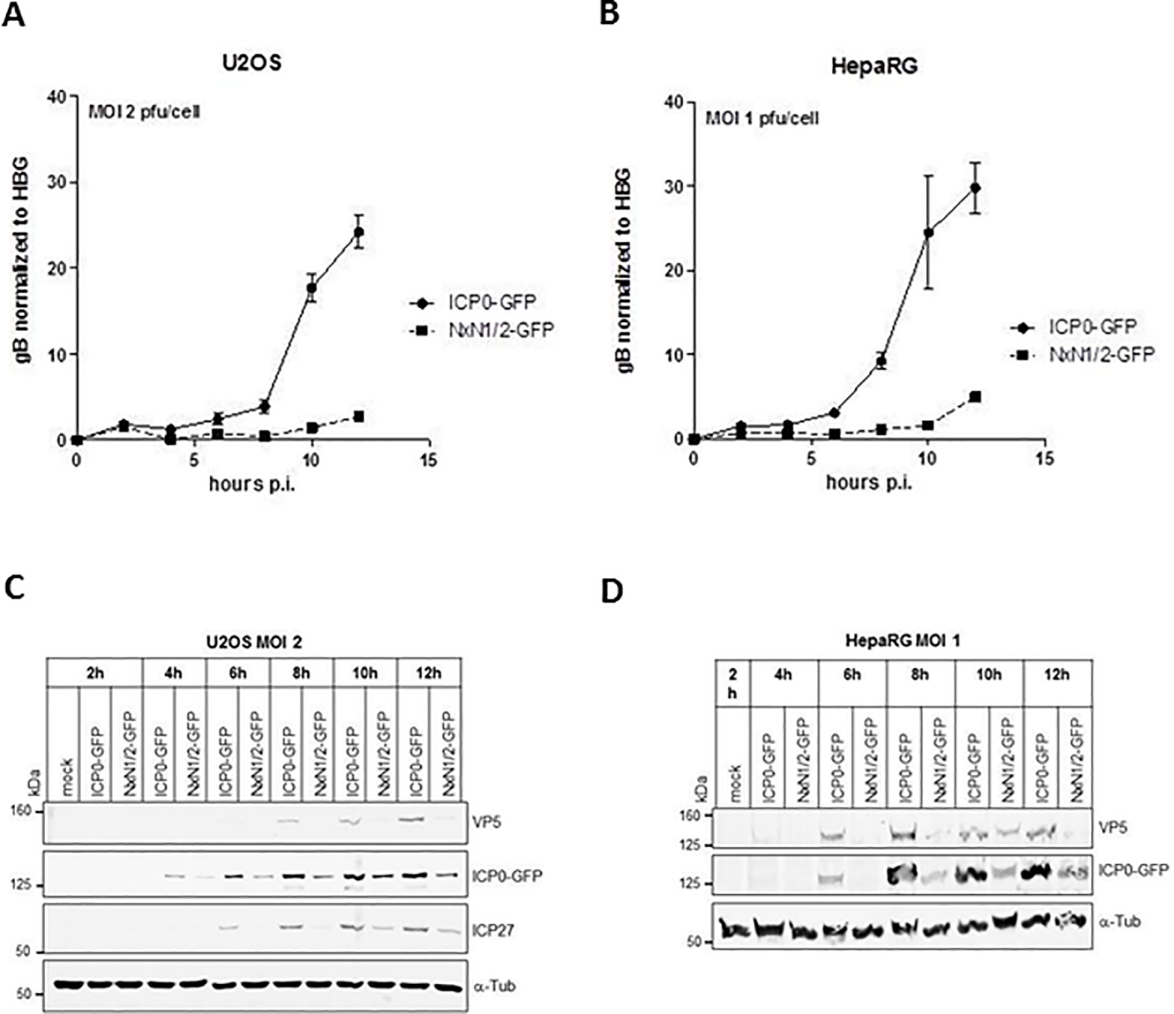
NxN1/2 mutation in ICP0 results in delayed and reduced vital DNA and protein synthesis. (**A**) U2OS cells, and (**B**) HepaRG cells were infected with HSV-2-ICP0-GFP (ICP0-GFP) or HSV-2-ICP0^NxN1/2^-GFP (NxN1/2-GFP) at an MOI of 2 or 1 pfu/cell, respectively. Infected cells were harvested at the indicated time points post infection (p.i.) for isolation of genomic and viral DNA. Viral DNA copies were determined by quantitative real-time PCR using oligonucleotide-primers and probes directed against the *UL27* gene encoding glycoprotein gB. Copy numbers were normalized to the sequence of the human single-copy beta-globin gene (HBG). (**C**) U2OS cells, and (**D**) HepaRG cells were infected with HSV-2-ICP0-GFP or HSV-2-ICP0^NxN1/2^-GFP at an MOI of 2 for up to 24 hours, or 1 pfu/cell for 12 hours, respectively. At the indicated time points, cells were harvested. Whole cell lysates were analyzed by SDS-PAGE and Western blot against the indicated proteins. Signals were quantified in S2 Fig.

Next, we compared DNA and protein expression *in vitro* mediated by the HSV-2^NxN1/2^-GFP and HSV-2-ICP0-GFP virus constructs. After infecting U2OS or HepaRG cells with both viruses at an MOI 1 or 2 pfu/cell, respectively, cell extracts were prepared at different time points post infection for subsequent analysis of viral DNA levels by quantitative real-time PCR, and viral protein expression by Western blotting. Viral DNA synthesis commenced shortly after infection (Fig 5A and 5B). However, starting at 5 hours post infection viral DNA copy numbers in HSV-2-ICP0^NxN1/2^-GFP infected cells were significantly lower than copy numbers produced by HSV-2-ICP0-GFP at each time point analyzed. (Fig 5A and 5B). This difference remained during the entire experimental period of 12 hours and became even more distinct as time progressed.

The relative attenuation of HSV-2-ICP0^NxN1/2^-GFP was also reflected at the level of viral *de novo* protein expression (Fig 5C and 5D). In immunoblot analyses, immediate-early gene synthesis was detected by ICP0- and ICP27-specific antibodies, while viral late gene expression was monitored detecting the major capsid protein VP5. Within the first 12 hours post infection protein abundance was lower in cells infected with the SIAH-1 binding-deficient virus mutant HSV-2-ICP0^NxN1/2^-GFP (Fig 5C and 5D). This was observed with both immediate early (ICP0 and ICP27) and late proteins (VP5), although synthesis of the latter was clearly more impaired (Fig 5C and 5D). The differences in viral protein expression became even more obvious when the quantified Western blot signals were normalized to the fluorescence intensity of the loading control α-tubulin (S7 Fig). Specifically, between 8 and 12 hours post infection all viral protein signals, including those of ICP0, were clearly higher in cells infected with wild-type virus than in cells infected with SIAH-1 binding-deficient virus (S7A and S7B Fig).

### Inactivation of the SIAH binding motif in ICP0 results in impaired HSV-2 replication in trigeminal ganglia and the brain stem *in vivo*

**Fig 6:**
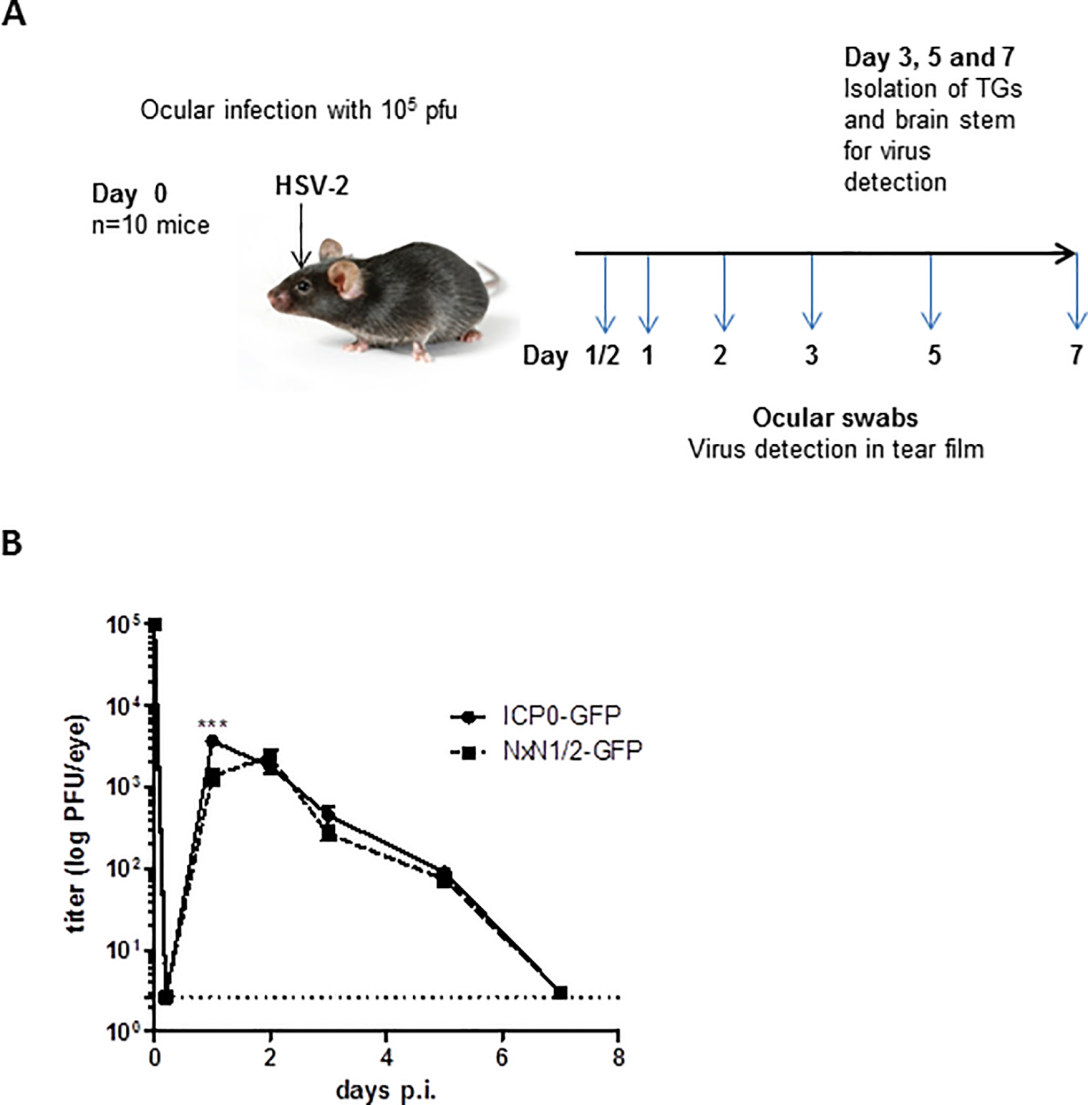
HSV-2 replication in the cornea of C57Bl/6J mice. (**A**) Schematic representation of the murine ocular HSV-2 infection model. Both corneas of anesthetized mice were scarified. Virus was placed on the eyes using 10^5^ pfu/eye. Ocular swabs were taken at the indicated time points post infection (p.i.). Animals were sacrificed at day 3, 5 and 7 post infection, and trigeminal ganglia (TG) and the brain stem were isolated for further analyses. (**B**) Ocular swabs from HSV-2-ICP0-GFP (ICP0-GFP) or HSV-2-ICP0^NxN1/2^-GFP (NxN1/2-GFP) infected animals taken at the indicated time points p.i. were analyzed for viral titers. For every experiment a group of n = 10 mice were infected and experiments were repeated four times. Each symbol represents the mean ± SEM of the titers, determined on Vero cell monolayers. The limit of detection is indicated by a dashed line. Statistical differences were calculated using two-way ANOVA with *** p< 0.001.

We next challenged C57Bl/6J mice via the ocular route (Fig 6A) [41,46]. After infection, viruses replicate in the eye, enter the innervating sensory neurons of the trigeminal ganglia (TG) and travel in a retrograde direction towards neuronal cell bodies and even further to the brain stem [4,5,47,48]. In our experimental set-up, 10^5^ plaque forming units (pfu) of the respective virus preparation (HSV-2-ICP0^NxN1/2^-GFP or HSV-2-ICP0-GFP) were applied to the cornea of anesthetized animals and virus replication was detected in the tear film obtained by ocular swabs, initially taken at 5 hours and at day 1, 2, 3, 5 and 7 post infection (Fig 6A). When comparing the viral load, particularly at 1 day post infection titers of the SIAH-1 binding-deficient mutant were significantly lower than the titers of the virus expressing ICP0-GFP protein (Fig 6B). Clearly, at later time points both viral constructs reached comparable titers (Fig 6B). At day 7 post infection, virus could no longer be detected in the tear film, indicating a complete clearance of virus in the cornea.

**Fig 7:**
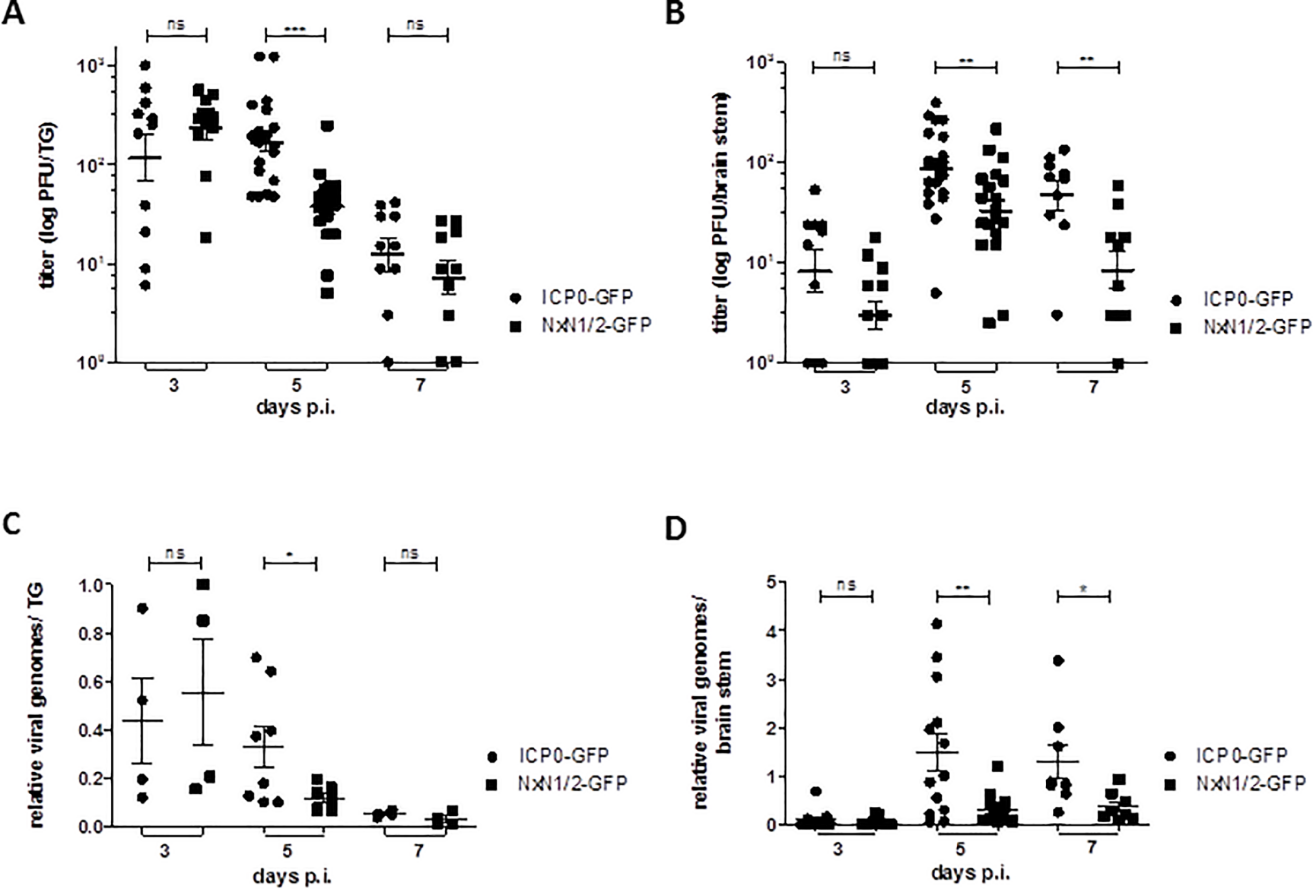
The NxN1/2 mutation in ICP0 leads to impaired replication in the TG and brain stem of C57Bl/6J mice. At day 3, 5 and 7 post infection (p.i.), (**A**) the trigeminal ganglia (TG), and (**B**) brain stem was extracted from HSV-2-ICP0-GFP and HSV-2-ICP0^NxN1/2^-GFP infected mice, homogenized and viral titers were determined as in Fig 6. Symbols represent individual animals; floating bars represent the mean ± SEM. Titer differences were determined by the Mann-Whitney test. (**C**) Half of the homogenized TG tissue from panel A, and (**D**) half of the tissue from panel B was lysed and total DNA was isolated to determine viral genome copy numbers. Oligonucleotide-primers specific for the HSV-2 *U*_*L*_*27* gene were used and copy numbers were normalized to murine β-actin sequences. Each symbol represents the relative genome number of an individual animal, relative to the genome copy number of a single HSV-2-ICP0-GFP infected mouse, which was set as 100%. Floating bars represent the mean ± SEM. Statistical differences were determined as above.

Following corneal infection, virus particles can be detected in the TG as early as 2 to 3 days post infection [49] and virus clearance commences at day 6 post infection [50]. Therefore, we analyzed virus dissemination to the innervating neurons, and virus replication in the infected tissue at 3, 5 and 7 days post infection by isolating and homogenizing the TG and brain stem of acutely infected mice (Fig 7). Half of the tissue samples were used for determining viral titers and the other half were saved for isolating DNA to quantify viral genome copy numbers by quantitative real-time PCR.

Titer measurements revealed that comparable amounts of viral wild-type and NxN1/2 mutant particles were detected in the TG at day 3 post infection (Fig 7A), suggesting that the ICP0:SIAH-1 interaction is not required for virus dissemination from the eye to the innervating neurons. However, on day 5 post infection titers of the NxN1/2 virus construct were significantly lower in the TG when compared to the titers reached by the ICP0 wild-type virus. This was also seen when the brain stem was analyzed (Fig 7B). In the TG, virus titers were clearly reduced at day 7 post infection and no significant difference was detected between the NxN1/2 mutant and the ICP0 wild-type construct; however comparably higher average virus titers were observed in the brain stem (Fig 7B).

Although less sensitive due to limited TG and brain stem samples, analysis of total viral DNA in these tissues confirmed the results above, showing significantly elevated levels of viral genome copies of the ICP0 wild-type virus as opposed to the NxN1/2 virus mutant, particularly in the brain stem (Fig 7C and 7D). These results suggest that the SIAH-binding deficient mutation significantly impairs HSV-2 replication in the TG and brain stem, thereby possibly affecting virus dissemination kinetics.

## Discussion

In a previous study we identified SIAH-1 as a host cell factor that specifically interacts with the viral regulatory protein ICP0 [30]. Here, we show that abolishing the ICP0:SIAH-1 interaction affects the HSV-2 phenotype during infection. In cell culture, the SIAH-binding deficient mutant synthesized significantly less DNA and protein, and virus growth was clearly attenuated compared to wild-type virus (Fig 5). In an animal model, HSV-2 carrying the SIAH-binding deficient mutation caused an acute infection in the murine eye and was able to spread to the innervating neurons (Fig 6B and Fig 7). However, the titers of the SIAH-1 binding-deficient virus detected in the TG and brain stem at day 5 post infection were much lower than the titers of wild-type virus (Fig 7A and 7B). Also, the viral DNA load in these tissues was clearly lower in case of the SIAH-1 binding-deficient virus (Fig 7C and 7D). Of note, the mouse model was considered a suitable system to study ICP0:SIAH-1 interaction, since, due to its evolutionary conservation, the murine genome encodes a SIAH-1 homolog called SIAH1A [51]. Human SIAH-1 and murine SIAH1A only differ in a single amino acid, and apparently fulfill identical functions [31].

The cellular/proteomic consequences evoked in the infected host cell by disrupting the interaction between ICP0 and SIAH-1 are currently not known. Both proteins belong to the RING-finger domain-containing family of E3 ubiquitin ligases, which induce proteasome-dependent degradation of various cellular factors [52,53]. For example, ICP0 and SIAH are both capable of mediating promyelocytic leukemia protein (PML) degradation [24,54,55]; PML structures are considered to be the sites where viral transcription and replication take place [56,57]. However, the dispersal of PML bodies appeared not to depend on the ICP0:SIAH-1 interaction. In fact, PML bodies were still degraded to comparable degrees in cells infected with either SIAH-1 binding-deficient virus or the ICP0 wild-type virus (data not shown). These results confirmed previous experiments obtained in cell cultures transiently transfected with ICP0 expression constructs [30]. However at this point, it cannot be completely ruled out that ICP0-mediated proteasomal degradation of other PML-unrelated cellular targets might be hampered when the ICP0:SIAH-1 interaction is compromised.

A single PxAxVxP-type binding motif for SIAH recognition is also located at amino acid positions 401–407 in ICP0 from Herpes Simplex Virus type 1 (HSV-1) [30]. This ICP0 region was previously analyzed for its ability to promote, for example, transcription from viral promoters or to affect HSV-1 growth. For instance, plasmids encoding ICP0 with a five aa insertion at residue 406 (thus disrupting the VxP motif), or deletion of the VxP motif, both displayed a reduced capability (7–22%) to induce transcription from the gD promoter in cooperation with ICP4 transcription [58], although the intracellular localization of both regulators, ICP0 and ICP4, was unaltered [59]. Furthermore, another independent study using promoter activation assays showed that deletion of the aa residues 393–448, or aa residues at positions 400–462 in HSV-1 ICP0 reduced its transcriptional activity to 34–81% of wild-type activity, depending on the reporter construct used [60]. Moreover, depending on MOI and cell type, an HSV-1 mutant encoding ICP0Δaa400-462 yielded only 4–17% of total viral particles compared to wild-type virus [61]. Finally, the aforementioned HSV-1 mutant containing a five aa insertion at ICP0 position 406 [58] was severely attenuated in BHK cells and displayed reduced protein synthesis, although in human fetal lung cells this defect was not as pronounced [25].

It may be conceivable that the mutant phenotypes described above can be explained, at least in part, by the lack of ICP0:SIAH interaction, thereby compromising ICP0’s ability to efficiently induce viral transcription, and in consequence, virus replication. This notion is in good accordance with the attenuated phenotype observed with our HSV-2-ICP0^NxN1/2^-GFP mutant virus. Although the RING domain probably constitutes the region of ICP0 most important for initiation and promotion of HSV infection [6,38,50], the inability of ICP0 to bind SIAH also proved to be detrimental to virus growth. Clearly, structural impairment of ICP0 could also result in a loss of ICP0 function. The two amino acid exchanges in our NxN1/2-mutant could result in small structural alterations, which could interfere with other ICP0 functions apart from SIAH-1 binding. It is noted, however, that localization of ICP0 during HSV-2 infection was unaffected by this mutation (data not shown).

So why does HSV-2 require ICP0 binding to SIAH-1 for efficient replication? One could envision three scenarios: i) SIAH-1 acquisition could expand ICP0’s target range in its role of degrading cellular proteins; ii) ICP0 could inactivate SIAH-1 by ubiquitination, or iii) ICP0 may function as a decoy to sequester SIAH-1 away from its own targets. Such mechanisms would increase the abundance of SIAH-1 target proteins, some of which might be required for efficient viral replication.

It was previously noticed that after cotransfection SIAH-1 is stabilized by ICP0, which may be explained by the inhibition of SIAH-1 auto-ubiquitination [30]. Altogether, interaction with ICP0 may have multiple consequences that could functionally inactivate SIAH-1 (for a model see S8 Fig): i) Both E3 RING ubiquitin ligases have the potential for mutual polyubiquitination; ii) ICP0 inhibits SIAH auto-ubiquitination; iii) both proteins interact in low-soluble aggregates; iv) ICP0 might inhibit ubiquitination of SIAH target proteins [30]. Although SIAH-1 appears to negatively affect the stability of ICP0 by ubiquitination [30], the net outcome of the ICP0:SIAH-1 interaction might be that SIAH-1 is captured by ICP0, which results in SIAH-1 depletion from the active cellular pool. Although SIAH ubiquitin ligases have so far not been regarded as a general intrinsic antiviral defense mechanism, their inactivation by ICP0 surely contributes to a cellular environment favorable to virus infection.

Of the many SIAH targets, some are involved in cellular pathways that might be crucial at different stages of infection. For example, SIAH’s involvement in DNA damage signaling [35,62] or in maintaining presynaptic proteostasis [63] may be of particular relevance for replication or the spread of a neurotropic DNA virus. An interesting speculation is driven by the observation that SIAH-1 mediates the polyubiquitination and degradation of the ELL2 protein, a crucial component of super-elongation complexes (SEC) [64]. SECs contain several transcription factors required for elongation by RNA polymerase II, and were originally identified by their roles in HIV-1 transcription and gene regulation through mixed lineage leukemia protein [65]. Interestingly, inhibition of SIAH-1 resulted not only in increased intracellular ELL2 levels, but also in increased transcription from the HIV-1 LTR promoter, which was shown to depend on functional SECs [64,66]. It is therefore tempting to speculate that interfering with SIAH-1 function is a viral strategy adopted by ICP0 to promote efficient HSV-2 gene expression at the level of transcriptional elongation. Future experiments may therefore focus on the effect of ICP0 on the abundance of SIAH-1 target proteins. These data might reveal valuable information about virus-induced changes in cellular protein interaction networks, and broaden the base for developing novel antiviral therapies.

## Supporting information

S1 Table Transfer plasmids for homologous recombination. (PDF)

S2 Table Viral titers on different cell lines at 48 hours post-infection. Passage 2 (P2) virus of the listed HSV-2 constructs was produced in BHK cells (see Methods). The P2 inoculum was subsequently titrated on U2OS, Vero and HepaRG cells to compare the growth efficiencies on different cell lines.
(PDF)

S1 Fig Analysis of HSV-2 ICP0 mutant constructs. Viral DNA was digested with HindIII and NcoI and subjected to Southern blot analyses using ICP0- (left panel) and GFP-specific (right panel) radioactively-labelled probes. Constructs marked in green were selected for further analysis.
(PDF)

S2 Fig HSV-2 *in vitro* growth curves. Vero cells were infected in duplicates with HSV‐2‐ICP0‐GFP (ICP0‐GFP) or HSV-2 MS (wt) using (**A**) an MOI of 0,01 pfu/cell or (**B**) an MOI of 1 pfu/cell. At the indicated time points, cells were harvested and titrated on Vero cell monolayers. Mean ± SD of viral titers (n = 2 per data point) were plotted as a function of time.
(PDF)

S3 Fig Ability of mutated ICP0 to degrade PML. Vero cells were infected with HSV‐2‐ICP0‐GFP or HSV‐2‐ICP0^NxN1/2^‐GFP at an MOI of 1 for up to 24 hours. Cells were harvested at the indicated time points and whole cell lysates were analyzed by SDS‐PAGE and Western blot using antibodies raised against the indicated proteins.
(PDF)

S4 Fig Analysis of cytoplasmic accumulation of ICP0. U2OS cells (2x10^5^) were infected with an MOI of 5 pfu/cell of the indicated virus construct. At 2, 4 and 6 h post infection, cells were fixed for 20 min with 4% PFA. Nuclei were stained with 2 μM Hoechst 33342 and plasma membrane structures with Alexa Fluor®647 wheat germ agglutinin (WGA). Localization of ICP0-GFP was visualized by confocal fluorescence microscopy using a Nikon Eclipse Ti-E system.
(PDF)

S5 Fig Absolute viral titers on different cell lines at 48 hours post-infection. Graphical comparison of viral titers as listed in S2 Table.
(PDF)

S6 Fig Analysis of plaque forming probability of HSV-2 constructs. Dilution series were performed to determine the relative plaque formation efficiency of the indicated HSV-2 constructs on Vero and HepaRG cells. Obtained titers were used to calculate the ratio of plaques on Vero cells to U2OS cells, and HepaRG cells to U2OS cells, independently for every HSV-2 construct. The titer on U2OS cells was arbitrarily set as 100%; wt, wild-type.
(PDF)

S7 Fig Quantification of protein expression in wild-type vs NxN1/2-GFP virus infected cells. (**A**) HepaRG cells were infected with the indicated constructs as described in Fig 5C and (**B**) U2OS cells were infected with the indicated constructs as described in Fig 5D. The fluorescent signal intensities of the secondary antibodies detecting the indicated viral proteins were quantified and normalized to the signal intensities of the loading control α-tubulin. Relative intensities are shown in pixels.
(PDF)

S8 Fig Schematic representation of the ICP0:SIAH-1 cross-talk. ICP0 and SIAH-1 are E3 ubiquitin ligases that are potentially able to ubiquitinate each other and, mostly in absence of a target protein, can also auto-ubiquitinate. It is hypothesized that ICP0 ubiquitinates SIAH-1 *in vivo* leading to the proteasomal degradation of the latter and to the stabilization of a so far unknown target protein that is needed for efficient progression of HSV-2 infection. When ICP0 auto-ubiquitinates or is ubiquinated by SIAH-1, it can be de-ubiquitinated by USP7. In turn, ICP0 can also ubiquitinate USP7, leading to the proteasomal degradation of the latter.
(PDF)
