# Supplementary material for: Interaction between the cellular E3 ubiquitin ligase SIAH-1 and the viral immediate-early protein ICP0 enables efficient replication of Herpes Simplex Virus type 2 in vivo
Source: PLoS One. 2018 Aug 6;13(8):e0201880. doi: 10.1371/journal.pone.0201880 (PMC6078308; doi:10.1371/journal.pone.0201880)
Supplement: S1 Table — (PDF) [file pone.0201880.s001.pdf]

**Transfer plasmid**	**Resulting virus**
pICP0hom-ICP0-GFP	HSV-2 -ICP0-GFP
pICP0hom-ICP0$^{NxN1/2}$-GFP	HSV-2 -ICP0$^{NxN1/2}$-GFP
pICP0hom-ICP0^{NxN1}-GFP	HSV-2 -ICP0^{NxN1}-GFP
pICP0hom-ICP0^{NxN2}-GFP	HSV-2 -ICP0^{NxN2}-GFP
