# Supplementary material for: Interaction between the cellular E3 ubiquitin ligase SIAH-1 and the viral immediate-early protein ICP0 enables efficient replication of Herpes Simplex Virus type 2 in vivo
Source: PLoS One. 2018 Aug 6;13(8):e0201880. doi: 10.1371/journal.pone.0201880 (PMC6078308; doi:10.1371/journal.pone.0201880)
Supplement: S2 Table — Passage 2 (P2) virus of the listed HSV-2 constructs was produced in BHK cells (see Methods). The P2 inoculum was subsequently titrated on U2OS, Vero and HepaRG cells to compare the growth efficiencies on different cell lines. (PDF) [file pone.0201880.s002.pdf]

	Titer (pfu/ml)		
**Virus**	**U2OS**	**Vero**	**HepaRG**
HSV-2 strain MS	2.8x10^7	3.5x10^7	n.d.
HSV-2-ICP0-GFP	1.85x10^8	1.9x10^8	4.9x10^7
HSV-2-ICP0$^{NxN1/2}$-GFP	1.53x10^8	1.5x10^8	6.0x10^7
HSV-2-GFP-ICP0$^{\Delta 19\text{-}162}$	1.5x10^7	3.0x10^5	n.d.
