# Supplementary material for: Interaction between the cellular E3 ubiquitin ligase SIAH-1 and the viral immediate-early protein ICP0 enables efficient replication of Herpes Simplex Virus type 2 in vivo
Source: PLoS One. 2018 Aug 6;13(8):e0201880. doi: 10.1371/journal.pone.0201880 (PMC6078308; doi:10.1371/journal.pone.0201880)
Supplement: S1 Fig — Viral DNA was digested with HindIII and NcoI and subjected to Southern blot analyses using ICP0- (left panel) and GFP-specific (right panel) radioactively-labelled probes. Constructs marked in green were selected for further analysis. (PDF) [file pone.0201880.s003.pdf]

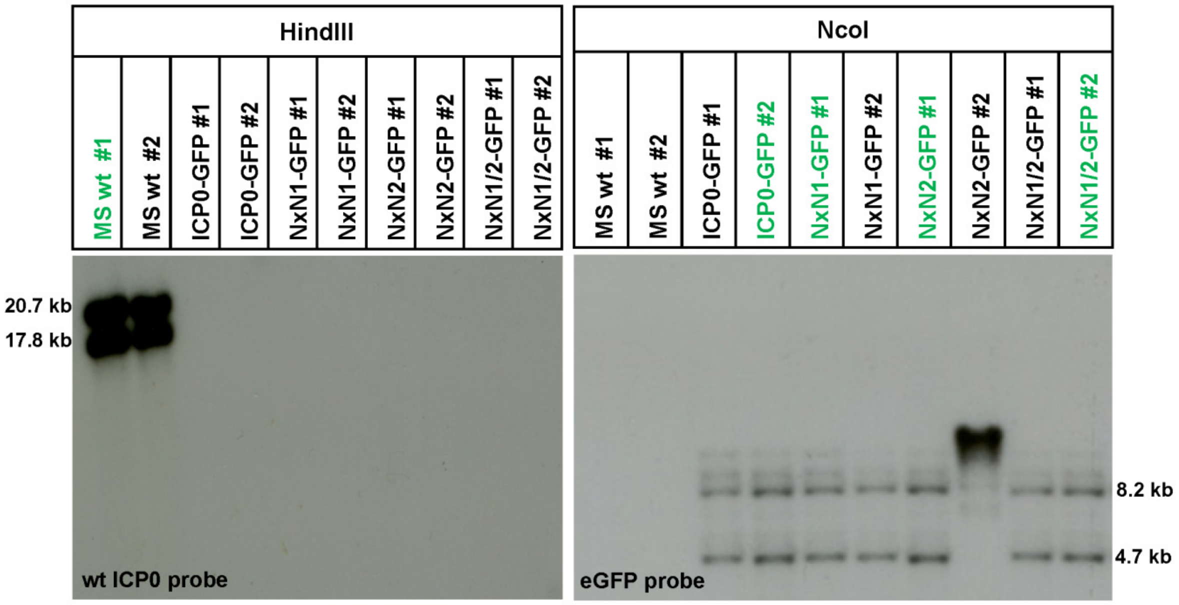
HindIII
MS wt #1
MS wt #2
ICP0-GFP #1
ICP0-GFP #2
NxN1-GFP #1
NxN1-GFP #2
NxN2-GFP #1
NxN2-GFP #2
NxN1/2-GFP #1
NxN1/2-GFP #2
20.7 kb
17.8 kb
wt ICP0 probe
NcoI
MS wt #1
MS wt #2
ICP0-GFP #1
ICP0-GFP #2
NxN1-GFP #1
NxN1-GFP #2
NxN2-GFP #1
NxN2-GFP #2
NxN1/2-GFP #1
NxN1/2-GFP #2
8.2 kb
4.7 kb
eGFP probe
