# Supplementary figures and images for: Interaction between the cellular E3 ubiquitin ligase SIAH-1 and the viral immediate-early protein ICP0 enables efficient replication of Herpes Simplex Virus type 2 in vivo
Source: PLoS One. 2018 Aug 6;13(8):e0201880. doi: 10.1371/journal.pone.0201880 (PMC6078308; doi:10.1371/journal.pone.0201880)

**A**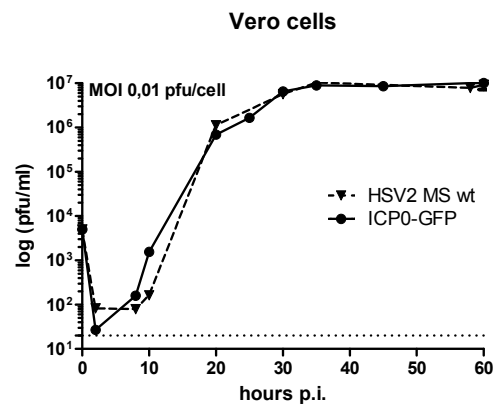**B**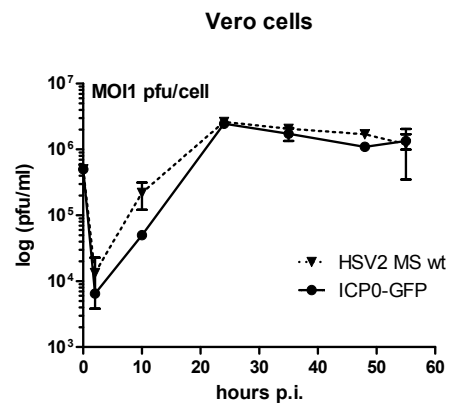

Supplement: S2 Fig — Vero cells were infected in duplicates with HSV‐2‐ICP0‐GFP (ICP0‐GFP) or HSV-2 MS (wt) using (A) an MOI of 0,01 pfu/cell or (B) an MOI of 1 pfu/cell. At the indicated time points, cells were harvested and titrated on Vero cell monolayers. Mean ± SD of viral titers (n = 2 per data point) were plotted as a function of time. (PDF) [file pone.0201880.s004.pdf]

Vero cells MOI 1

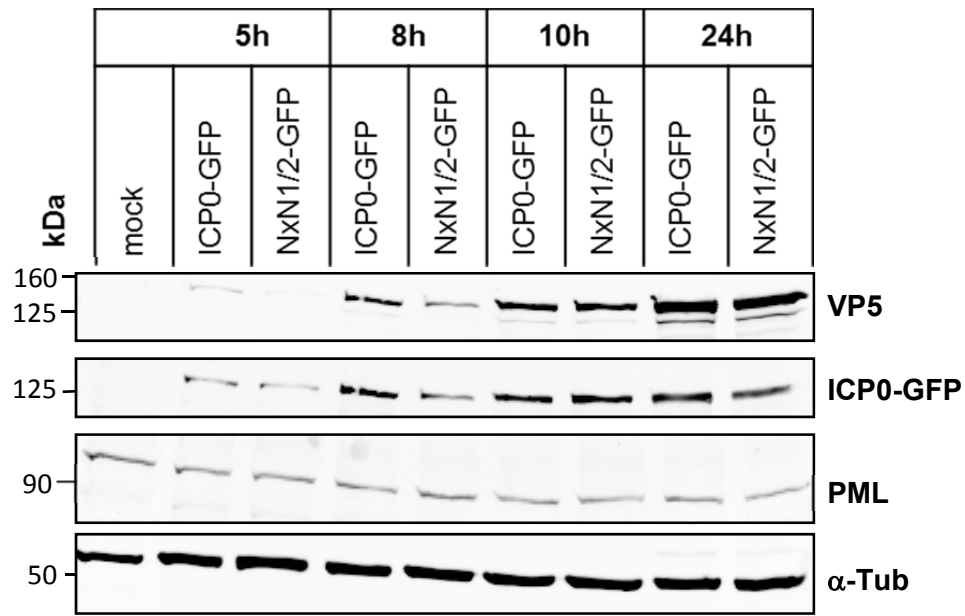

Supplement: S3 Fig — Vero cells were infected with HSV‐2‐ICP0‐GFP or HSV‐2‐ICP0NxN1/2‐GFP at an MOI of 1 for up to 24 hours. Cells were harvested at the indicated time points and whole cell lysates were analyzed by SDS‐PAGE and Western blot using antibodies raised against the indicated proteins. (PDF) [file pone.0201880.s005.pdf]

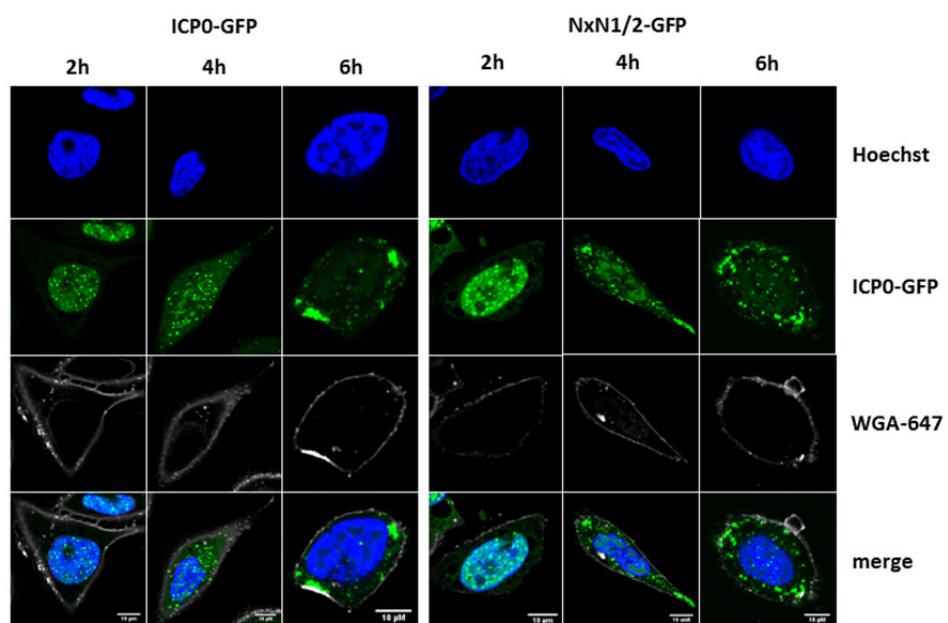

Supplement: S4 Fig — U2OS cells (2x10^5^) were infected with an MOI of 5 pfu/cell of the indicated virus construct. At 2, 4 and 6 h post infection, cells were fixed for 20 min with 4% PFA. Nuclei were stained with 2 μM Hoechst 33342 and plasma membrane structures with Alexa Fluor®647 wheat germ agglutinin (WGA). Localization of ICP0-GFP was visualized by confocal fluorescence microscopy using a Nikon Eclipse Ti-E system. (PDF) [file pone.0201880.s006.pdf]

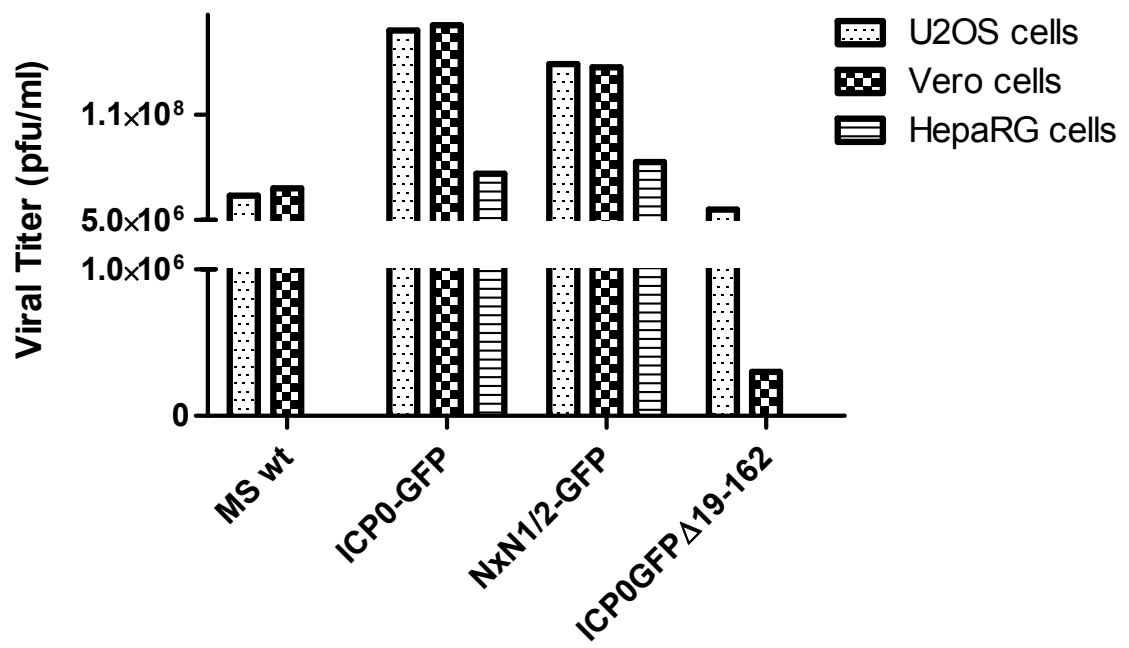

Supplement: S5 Fig — Graphical comparison of viral titers as listed in S2 Table. (PDF) [file pone.0201880.s007.pdf]

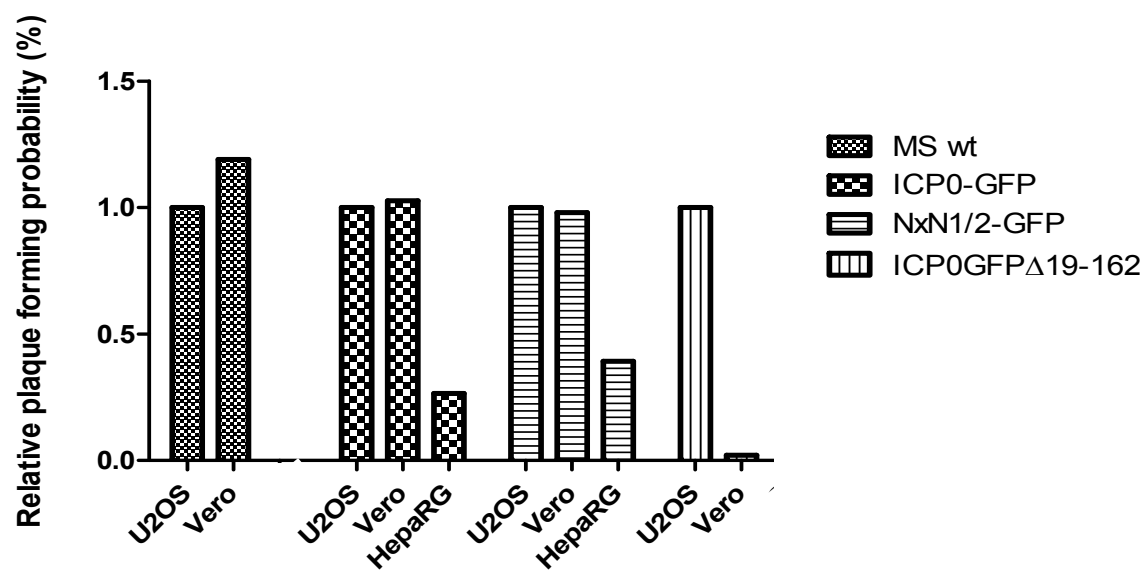

Supplement: S6 Fig — Dilution series were performed to determine the relative plaque formation efficiency of the indicated HSV-2 constructs on Vero and HepaRG cells. Obtained titers were used to calculate the ratio of plaques on Vero cells to U2OS cells, and HepaRG cells to U2OS cells, independently for every HSV-2 construct. The titer on U2OS cells was arbitrarily set as 100%; wt, wild-type. (PDF) [file pone.0201880.s008.pdf]

**A**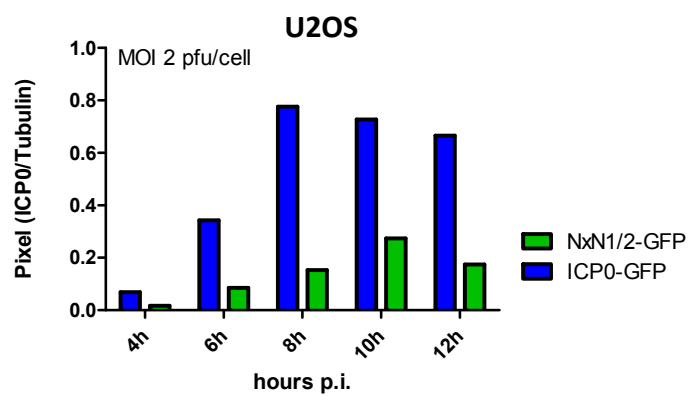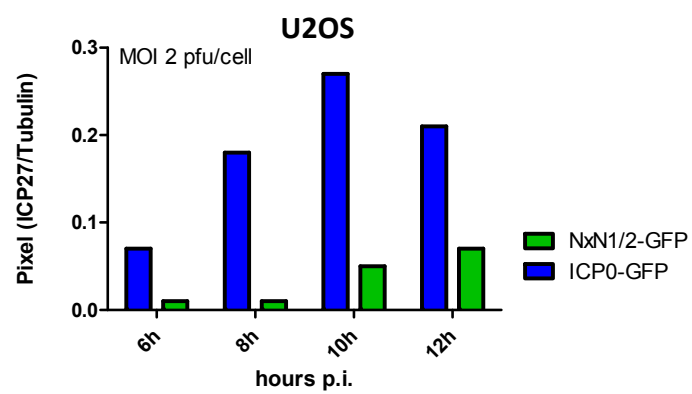**B**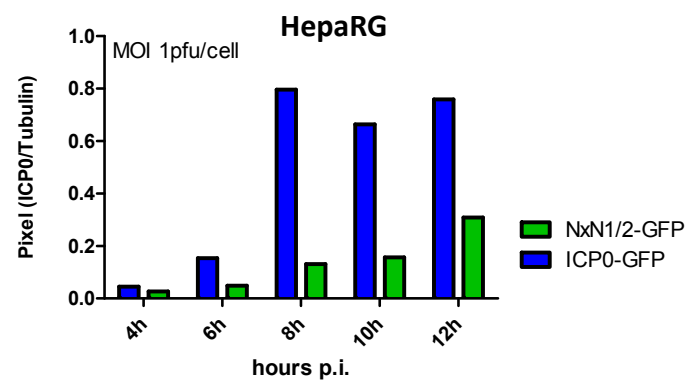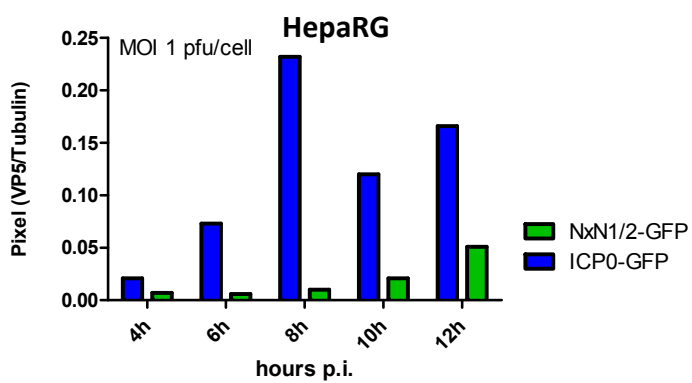

Supplement: S7 Fig — (A) HepaRG cells were infected with the indicated constructs as described in Fig 5C and (B) U2OS cells were infected with the indicated constructs as described in Fig 5D. The fluorescent signal intensities of the secondary antibodies detecting the indicated viral proteins were quantified and normalized to the signal intensities of the loading control α-tubulin. Relative intensities are shown in pixels. (PDF) [file pone.0201880.s009.pdf]

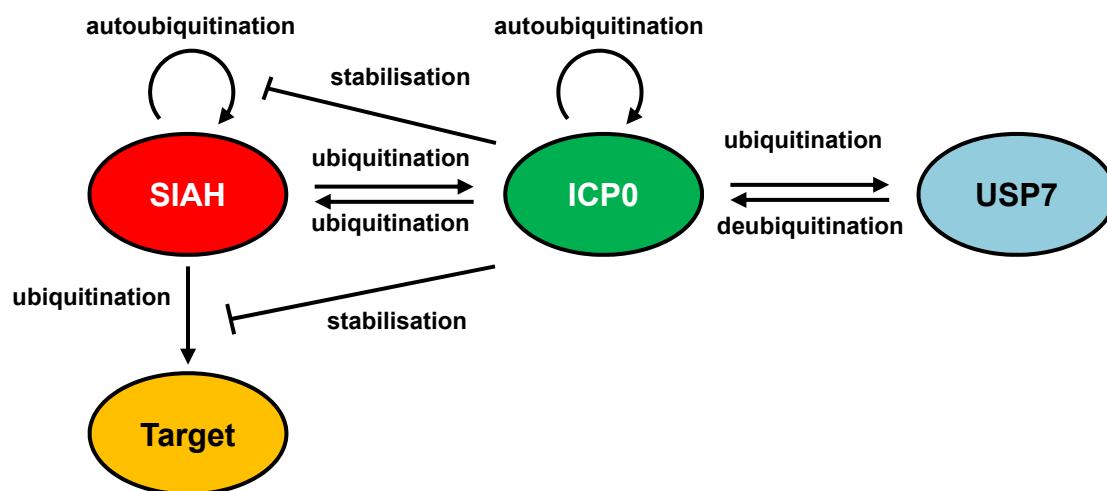

Supplement: S8 Fig — ICP0 and SIAH-1 are E3 ubiquitin ligases that are potentially able to ubiquitinate each other and, mostly in absence of a target protein, can also auto-ubiquitinate. It is hypothesized that ICP0 ubiquitinates SIAH-1 in vivo leading to the proteasomal degradation of the latter and to the stabilization of a so far unknown target protein that is needed for efficient progression of HSV-2 infection. When ICP0 auto-ubiquitinates or is ubiquinated by SIAH-1, it can be de-ubiquitinated by USP7. In turn, ICP0 can also ubiquitinate USP7, leading to the proteasomal degradation of the latter. (PDF) [file pone.0201880.s010.pdf]
